# RECIST and iRECIST criteria for the evaluation of nivolumab plus ipilimumab in patients with microsatellite instability-high/mismatch repair-deficient metastatic colorectal cancer: the GERCOR NIPICOL phase II study

**DOI:** 10.1136/jitc-2020-001499

**Published:** 2020-11-03

**Authors:** Romain Cohen, Jaafar Bennouna, Aurélia Meurisse, Christophe Tournigand, Christelle De La Fouchardière, David Tougeron, Christophe Borg, Thibault Mazard, Benoist Chibaudel, Marie-Line Garcia-Larnicol, Magali Svrcek, Dewi Vernerey, Yves Menu, Thierry André

**Affiliations:** 1 Sorbonne University, Department of Medical Oncology, Saint-Antoine Hospital, APHP, Paris, France; 2 Department of Medical Oncology, University Hospital of Nantes, Nantes, France; 3 Department of Oncology, Besançon University Hospital, Methodology and Quality of Life Unit, Besançon, France; 4 Department of Gastroenterology and Digestive Oncology, Henri Mondor University Hospital, APHP, Creteil, France; 5 Department of Medical Oncology, Centre Léon Bérard, Lyon, France; 6 Department of Gastroenterology, Poitiers University Hospital and University of Poitiers, Poitiers, France; 7 Department of Medical Oncology, University Hospital of Besançon, Besançon, France; 8 Department of Medical Oncology, Institut de Recherche en Cancérologie de Montpellier (IRCM), INSERM, Montpellier University, INSERM U1194, Montpellier, France; 9 Department of Medical Oncology, Franco-British Hospital, Fondation Cognacq-Jay, Levallois-Perret, France; 10 Multidisciplinary Group in Oncology (GERCOR), Paris, France; 11 Sorbonne University, Department of Pathology, Saint-Antoine Hospital, AP-HP, Paris, France; 12 Sorbonne University, Department of Radiology, Saint-Antoine Hospital, AP-HP, Paris, France

**Keywords:** immunotherapy, CTLA-4 antigen, gastrointestinal neoplasms, programmed cell death 1 receptor

## Abstract

**Background:**

Immune checkpoint inhibitors (ICIs) are highly effective in patients with microsatellite instability/mismatch repair-deficient (MSI/dMMR) metastatic colorectal cancer (mCRC). Response Evaluation Criteria in Solid Tumors (RECIST) 1.1 criteria may underestimate response to ICIs due to the pseudoprogression phenomenon. The GERCOR NIPICOL phase II study aimed to evaluate the frequency of pseudoprogressions in patients with MSI/dMMR mCRC treated with nivolumab and ipilimumab.

**Methods:**

Patients with MSI/dMMR mCRC previously treated with fluoropyrimidines, oxaliplatin, and irinotecan with/without targeted therapies received nivolumab 3 mg/kg plus ipilimumab 1 mg/kg every 3 weeks for four cycles then nivolumab 3 mg/kg every 2 weeks until progression or a maximum of 20 cycles. Computed tomography scan tumor assessments were done every 6 weeks for 24 weeks and then every 12 weeks. The primary endpoint was disease control rate at 12 weeks according to RECIST 1.1 and iRECIST by central review.

**Results:**

Of 57 patients included between December 2017 and November 2018, 48.0% received ≥3 prior lines of chemotherapy, 18.0% had *BRAF*^V600E^ mutation, and 56.0% had Lynch syndrome-related cancer. Seven patients (12.0%) discontinued treatment due to adverse events; one died due to a treatment-related adverse event. The disease control rate (DCR) at 12 weeks was 86.0% with RECIST 1.1% and 87.7% with iRECIST. Two pseudoprogressions (3.5%) were observed, at week 6 and at week 36, representing 18% of patients with disease progression per RECIST 1.1 criteria. With a median follow-up of 18.4 months, median progression-free survival (PFS) and overall survival (OS) were not reached. The 12-month PFS rate was 72.9% with RECIST 1.1% and 76.5% with iRECIST. The 12-month OS rate was 84%. Overall response rate was 59.7% with both criteria. *RAS*/*BRAF* status, sidedness, Lynch syndrome, and other baseline parameters were not associated with PFS.

**Conclusion:**

Pseudoprogression is rare in patients with MSI/dMMR mCRC treated with nivolumab and ipilimumab. This combined ICI therapy confirms impressive DCR and survival outcomes in these patients.

**Trial registration number:**

NCT03350126.

## Introduction

Microsatellite instability (MSI) is caused by the deficiency of the DNA mismatch repair (MMR) system, resulting from a germline mutation in *MMR* genes (Lynch syndrome) or from an epigenetic extinction of *MLH1* gene (sporadic cases).^1^ Sporadic MSI/MMR-deficient (dMMR) colorectal cancers (CRCs) are frequently associated with the *BRAF*^V600E^ mutation, a well-known negative prognostic factor. Approximately 5% of metastatic CRC (mCRC) are MSI/dMMR and one-third of them are *BRAF*^V600E^-mutated.^2^

Tumors with MSI/dMMR are characterized by a high tumor mutational burden, with highly immunogenic neoantigens arising from frameshift mutation and by a high infiltration of cytotoxic T lymphocytes. The upregulation of immune checkpoints by tumor cells protects them from this hostile microenvironment.^3 4^ Immune checkpoint inhibitors (ICIs) such as anti-programmed death 1 (PD1) or anti-programmed death ligand 1 (PD-L1) monoclonal antibodies, alone or in combination with anti-CTLA-4 agents, have demonstrated high clinical activity with durable responses in patients with MSI/dMMR mCRC.^5–9^ The KEYNOTE-177 phase III study showed a significant improvement of progression-free survival (PFS) with first-line pembrolizumab compared with standard-of-care chemotherapy plus targeted therapy.^10^

The initial effect of ICIs on tumor can include an increase of tumor diameter due to the immune cell infiltration. Conventional criteria for the evaluation of treatment responses, namely Response Evaluation Criteria in Solid Tumors (RECIST) 1.1, fail to identify a phenomenon of pseudoprogression and may falsely conclude that there is disease progression (PD). These criteria have been shown to underestimate the benefits of pembrolizumab in terms of overall response rate and PFS in approximately 15% of patients with melanoma.^11 12^ Consequently, modified RECIST 1.1 for immune-based therapeutics (iRECIST) have been developed, requiring the confirmation of PD to rule out or confirm pseudoprogression.^13 14^ Although pseudoprogression is well recognized and reported in up to 10% of patients with melanoma or lung cancer,^15–18^ its frequency in patients with MSI/dMMR treated with ICIs has never been evaluated. This knowledge is of great value, though, given the negative consequences of either discontinuation of an effective treatment or maintenance of an ineffective drug beyond PD. Therefore, we designed the GERCOR NIPICOL phase II study to evaluate RECIST1.1 and iRECIST criteria in patients with MSI/dMMR mCRC treated with the nivolumab and ipilimumab combination.

## Patients and methods

### Study design and population

This a single-arm, open-label, multicenter phase II study (NIPICOL) was designed (GERCOR) to evaluate disease control rate (DCR) by RECIST and iRECIST at 12 weeks in patients with MSI/dMMR mCRC. Patients were recruited from eight French hospitals. Eligible patients were ≥18 years old, had histologically confirmed mCRC locally assessed as MSI/dMMR, measurable disease per RECIST 1.1 criteria, and an Eastern Cooperative Oncology Group performance status (ECOG PS) of 0 or 1. Patients developed PD or were intolerant to approved standard therapies (fluoropyrimidine, oxaliplatin, irinotecan, antiangiogenics, and antiepidermal growth factor receptor agents if their tumors harbored wild-type *RAS*/*RAF*). Eligible patients had absolute neutrophil count ≥1500 cells per mm³, platelet count ≥100 x 10^9^/L, hemoglobin ≥9 g/dL, serum creatinine level <150 µM, aspartate aminotransferase and alanine aminotransferase ≤3 × upper limit of normal (ULN; or ≤5 × ULN in the case of known liver metastases), alkaline phosphatase <5 × ULN, and total bilirubin ≤1.5 × ULN. Main exclusion criteria included: prior treatment with an anti-PD1, anti-PD-L1, anti-CTLA-4, or other agent targeting T-cell co-stimulation or immune checkpoint pathways, conditions requiring corticosteroids (prednisone equivalents >10 mg/day) or other immunosuppressive medication within 2 weeks before starting treatment, other serious or uncontrolled medical disorders, active brain or leptomeningeal metastases, or prior malignancy within the previous 5 years except for cured select localized cancers.

### Procedures

Patients received nivolumab 3 mg/kg intravenously over 60 min and ipilimumab 1 mg/kg intravenously over 90 min every 3 weeks for four cycles (induction phase) and then nivolumab 3 mg/kg intravenously every 2 weeks until PD, discontinuation because of toxicity, death, withdrawal of consent, or for a maximum of 20 infusions, equivalent to 1 year of therapy. Dose modifications were not permitted.

### Outcomes

The primary endpoint was DCR at 12 weeks according to RECIST 1.1 and iRECIST criteria by central review. DCR was defined as the number of patients with stable disease (SD), partial response (PR), or complete response divided by the total number of patients. Secondary endpoints were safety, PFS, overall survival (OS; time from the first dose to death, whatever the cause), and association between baseline clinicopathological parameters and PFS. PFS by RECIST 1.1 was calculated from the first ICI infusion to the first documented PD or death resulting from any cause, whichever occurred first. PFS by iRECIST was calculated from the first dose to the first documented PD with subsequent confirmation or death resulting from any cause, whichever occurred first.

### Assessments

Baseline radiologic tumors assessments were assessed ≤28 days before the first dose (baseline) of treatment followed by every 6 weeks for 24 weeks and every 12 weeks thereafter by CT. RECIST and iRECIST evaluations were centrally reviewed by one radiologist (YM). Pseudoprogression was defined as an unconfirmed PD according to iRECIST.^14^ If pseudoprogression was suspected, the same images were independently blindly reviewed by a second radiologist. In case of discrepancy, a final decision was reached by consensus. Safety was assessed per National Cancer Institute Common Terminology Criteria for Adverse Events (NCI CTCAE) V.4.0.

The MSI/dMMR status was evaluated before screening by local laboratories and defined by high level of MSI by PCR testing (two instable markers or more; with recommended panel including: BAT25, BAT26, NR21, NR24, and NR27) and/or loss of expression of minimum one MMR protein by immunohistochemistry (testing of four MMR proteins: MLH1, PMS2, MSH2, and MSH6). Pathological and molecular biology reports were reviewed by the study sponsor prior to patients’ inclusion. Tumor samples (archival or fresh biopsy specimen from primary or metastatic lesions) were collected for central laboratory confirmation of the MSI/dMMR status (Saint-Antoine Hospital, Paris, France) and additional translational studies.

Tumors were considered related to Lynch syndrome in case of known MMR gene germline mutation, loss of expression of MSH2 and/or MSH6, or loss of expression of MLH1, and/or PMS2 without *BRAF*^V600E^ mutation nor hypermethylation of *MLH1* promoter. Tumors with loss of MLH1 and/or PMS2 harboring *BRAF*^V600E^ mutation and/or a hypermethylation of *MLH1* promoter were considered as sporadic. In all other cases, Lynch syndrome status was defined as unknown.^19^

### Statistical analysis

The study was designed considering an estimated 12-week DCR of 70% with RECIST 1.1. Using iRECIST, a 12 week DCR of 85% was expected (H1: alternative hypothesis), whereas a 12-week DCR of 70% was considered as uninteresting (H0: null hypothesis). According to a A'Hern single-stage phase II design^20 21^ with a one-sided 5% type I error and a power of 80%, 49 evaluable patients were needed for the analysis of the primary endpoint. Considering a minimal 15% rate of patients not evaluable for the primary endpoint, 57 patients were planned to be included. Statistical analyses were performed on an intention-to-treat basis.

Continuous and categorical variables were described by medians (IQR) and frequencies (percentage), respectively. The median PFS and OS, and the proportion of patients meeting these endpoints at specific time points were estimated by the Kaplan-Meier method. The 95% CI were calculated using log-log transformation. Median follow-up was calculated by the reverse Kaplan-Meier method.

Cox proportional-hazard models were used to estimate HRs and their 95% CIs for baseline factors associated with PFS. The association was evaluated in a prespecified exploratory analysis using the univariate Cox model and then parameters with p values of <0.1 were entered into a final multivariate Cox regression model.

A waterfall plot illustration was used to present the best percentage change in target lesion size from baseline during the first year of treatment. Duration of treatment and response in patients with at least one evaluation with SD or better were summarized in a swimmer plot. All analyses were performed using SAS V.9.4 (SAS Institute).

## Results

### Population

Between December 2017 and November 2018, 57 patients with MSI/dMMR mCRC were enrolled. Table 1 shows baseline clinical and pathological characteristics. Most patients were male (52.6%), with ECOG PS of 1 (64.9%), right-sided tumors (54.4%), two metastatic sites or more (71.9%). Twenty-eight (49.1%) and 10 (17.5%) patients’ tumors were *RAS*-mutated and *BRAF*^V600E^-mutated, respectively. Overall, 47.4% of patients were treated with ≥3 prior lines of treatment, including fluoropyrimidines (100.0%), oxaliplatin (100.0%), irinotecan (96.5%), antiangiogenics (57.9%), and anti-epidermal growth factor receptors (45.6%; table 1).

**Table 1:** Patient baseline characteristics

Characteristics	N=57
Age (years), median (Q1–Q3)	56.5 (45.8–63.8)
Sex, n (%)	
Male	30 (52.6)
Female	27 (47.4)
ECOG PS, n (%)	
0	20 (35.1)
1	37 (64.9)
Primary tumor location, n (%)	
Right-sided	31 (54.4)
Left-sided	25 (43.9)
Right and left location	1 (1.8)
Mutation status, n (%)	
*RAS*/*RAF* wild type	19 (33.3)
*RAS* mutation	28 (49.1)
*BRAF* mutation	10 (17.5)
Origin of MMR deficiency, n (%)	
Lynch-related	32 (56.1)
Known germline mutation	19 (59.4)
Sporadic	16 (28.1)
Unknown	9 (15.8)
Number of metastatic sites, n (%)	
1	16 (28.1)
2	25 (43.9)
>2	16 (28.1)
Number of prior lines, n (%)	
1	5 (8.8)
2	24 (42.1)
>2	27 (47.4)
Missing	1 (1.8)
Prior treatments, n (%)	
5-FU/capecitabine	57 (100.0)
Oxaliplatin	57 (100.0)
Irinotecan	55 (96.5)
Trifluridine/tipiracil	4 (7.0)
Regorafenib	5 (8.8)
Bevacizumab/aflibercept	33 (57.9)
Cetuximab/panitumumab	26 (45.6)

ECOG PS, The Eastern Cooperative Oncology Group Performance Status; 5-FU, 5-fluorouracil; MMR, DNA mismatch repair.

The database was locked on December 11, 2019, with a median follow-up of 18.1 months (95% CI 14.1 to 19.2). A total of 36 patients (63.2%) completed the predefined 1-year duration of treatment, five patients died from disease-related event during the induction phase (12 weeks). Other reasons of treatment discontinuation were progressive disease (n=8), adverse event (n=7) including one toxic death, and a patient’s wish (n=1).

### Safety

Thirty-two patients (56.1%) experienced grade ≥3 adverse events. Grade 3–5 treatment-related adverse events (TRAEs) were reported for 17 patients (29.8%), with one patient who died from septic shock while being treated with corticosteroid for a potentially immune-related hepatitis (table 2). Most frequent grade 3–5 TRAEs were increased transaminases (8.8%), increased serum lipase level (7.0%), diarrhea (3.5%), and fatigue (3.5%). Endocrine-related TRAEs included grade 3 adrenal insufficiency (n=1; 1.8%), grade 3 diabetes (n=1; 1.8%), grade 2 hypothyroidisms (n=3; 5.3%), and grade 2 hyperthyroidism (n=1; 1.8%). One patient was diagnosed with grade 3 sarcoidosis and one with grade 2 uveitis.

**Table 2:** Treatment-related adverse events

	N=57 (%)	
Treatment-related adverse events		
All grade	49 (86.0)	
Grade 3–5	17 (29.8)	
That led to discontinuation	7 (12.3)	
	**Grade 3–5**	**All grade**
Aspartate aminotransferase increase	5 (8.8)	6 (10.5)
Alanine aminotransferase increase	4 (7.0)	7 (12.3)
Lipase increase	4 (7.0)	6 (10.5)
Fatigue	2 (3.5)	26 (45.6)
Diarrhea	2 (3.5)	20 (35.1)
Anemia	1 (1.8)	11 (19.3)
Mucositis	1 (1.8)	6 (10.5)
Anorexia	1 (1.8)	4 (7.0)
Fever	1 (1.8)	3 (5.3)
Thrombopenia	1 (1.8)	3 (5.3)
Acute kidney injury	1 (1.8)	1 (1.8)
Diabetes	1 (1.8)	0 (0.0)
Gastritis	1 (1.8)	0 (0.0)
Pulmonary sarcoidosis	1 (1.8)	0 (0.0)
Creatine phosphokinase increase	1 (1.8)	0 (0.0)
Adrenal insufficiency	1 (1.8)	3 (5.3)
Hyperthyroidism	0 (0.0)	8 (14.0)
Hypothyroidism	0 (0.0)	6 (10.5)

### Efficacy

For eligible patients (n=57), the 12-week DCR was 86.0% by RECIST 1.1.% and 87.7% by iRECIST (central review; kappa coefficient=0.92, 95% CI 0.77 to 1.0 with no significant difference). Three patients died from disease-related cause prior to any tumor assessment and were considered as progressive. Five patients experienced PD at the first CT scan evaluation (week 6). Of these, two died from disease-related cause before the second CT scan and were considered as confirmed PD, two (who continued treatment) had confirmed PD on the subsequent CT scan at 12 weeks, and one had pseudoprogression. At 12 weeks, 2 (3.5%) CR and 18 (31.6%) PR were observed (table 3; online supplemental file 1).

10.1136/jitc-2020-001499.supp1 Supplementary data

**Table 3:** Tumor response at 12 weeks and overall best observed response per RECIST 1.1 criteria

	At 12 weeks N=57	Overall best response N=57
Complete response, n (%)	2 (3.5)	11 (19.3)
Partial response, n (%)	18 (31.6)	23 (40.4)
Objective response rate, %	35.1	59.6
Stable disease, n (%)	29 (50.9)	17 (29.8)
Progressive disease, n (%)	5 (8.8)	3 (5.3)
Confirmed progressive disease per iRECIST criteria	4 (7.0)	2 (3.5)
Pseudoprogression per iRECIST criteria	1 (1.8)	1 (1.8)
Non-evaluable, n (%)	3 (5.3)	3 (5.3)
Disease control rate per RECIST1.1/iRECIST, %	86.0/87.7	89.5/91.2

RECIST, Response Evaluation Criteria in Solid Tumors.

Overall, two pseudoprogressions were observed during follow-up (out of 57, 3.5%). The first pseudoprogression occurred at week 6 in a 68-year-old man treated for peritoneal metastases from a MLH1/PMS2-negative, MSI, *RAS*/*RAF* wild-type right-sided mucinous colon carcinoma. Study treatment was maintained, and the patient experienced PR after 11 months of treatment. The second pseudoprogression occurred at week 36 in a 47-year-old woman with a *KRAS*-mutated, MSI, MSH2/MSH6-negative mucinous, rectal carcinoma harboring mesenteric lymph node metastases. Best observed response was SD. Study treatment was interrupted in this patient 2 months after pseudoprogression due to grade 3 diarrhea. Both patients were alive and free of progression 22 and 27 months after treatment initiation, respectively.

**Figure 1:**
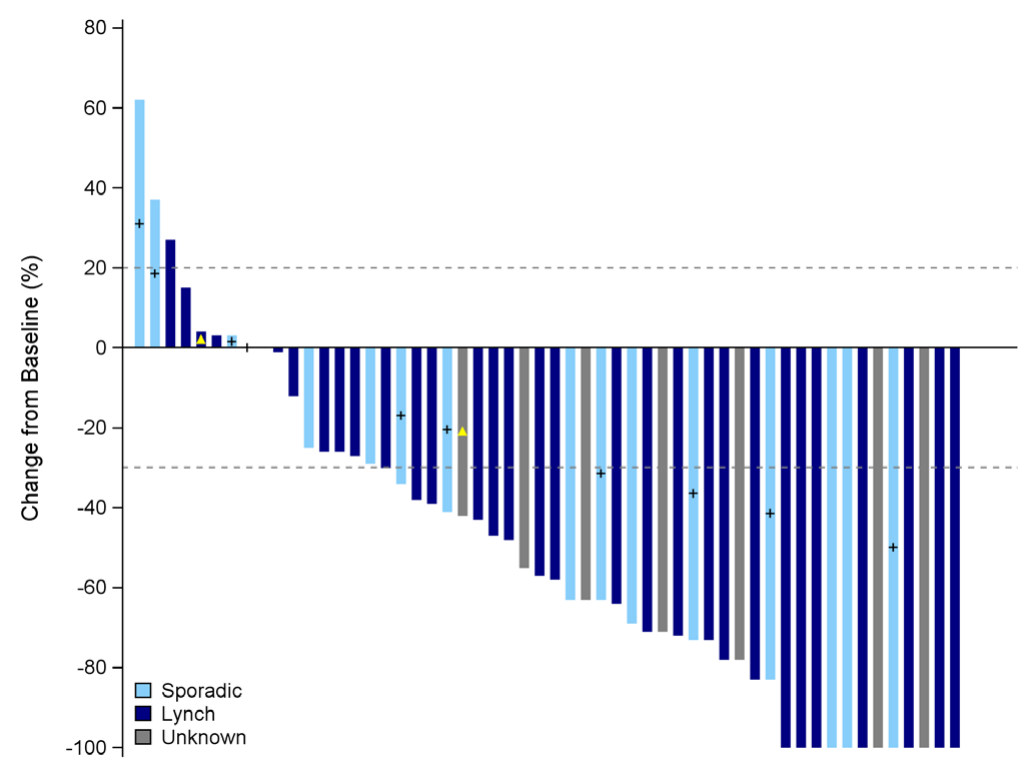
Best percentage change in target lesion size from baseline by central review. + sign: BRAF-mutated tumors, yellow triangle: pseudo-progression.

**Figure 2:**
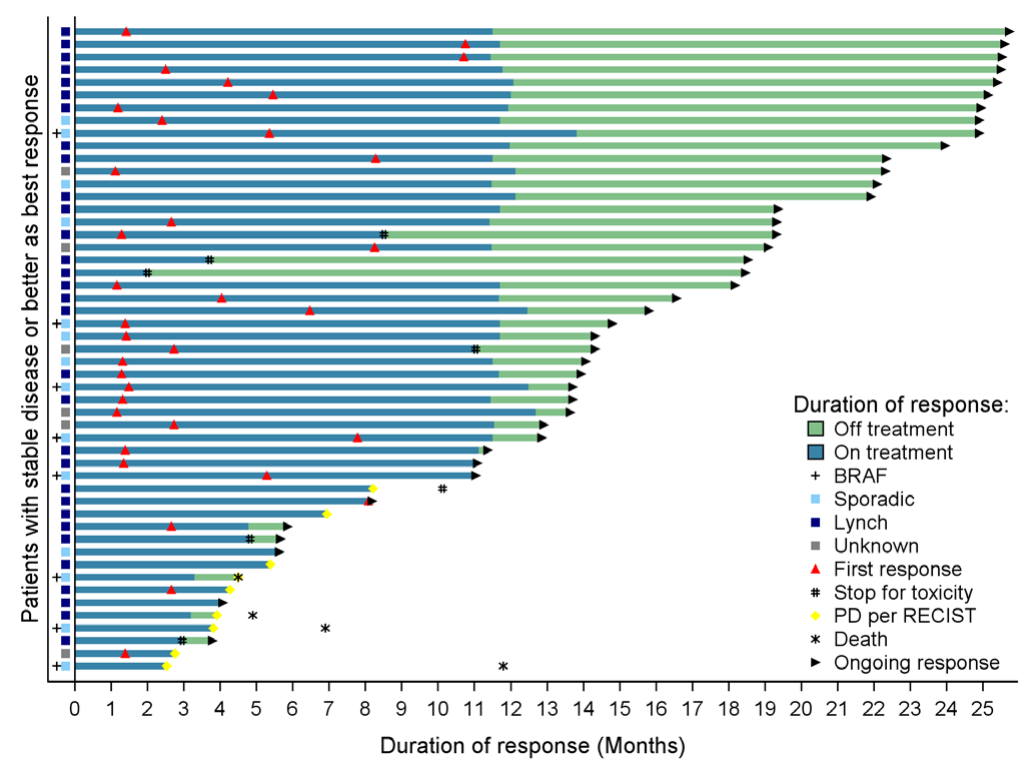
Duration of treatment and response in patients with stable disease or better disease control per RECIST v1.1 criteria. PD, disease progression; RECIST, Response Evaluation Criteria in Solid Tumors.

**Figure 3:**
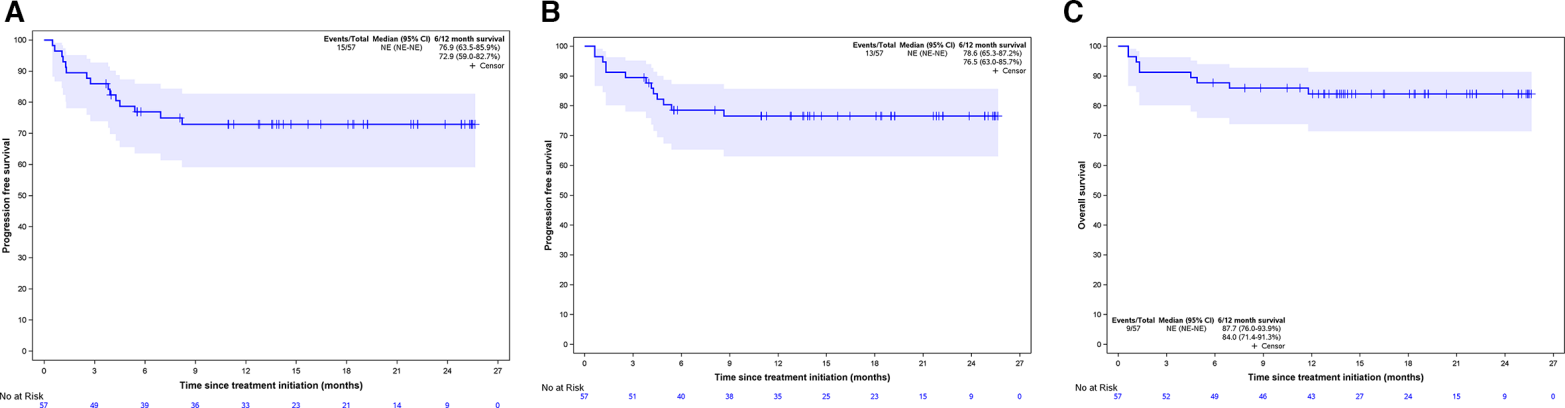
Kaplan-Meier survival curves of progression-free survival per RECIST v1.1 (A), per iRECIST (B) and overall survival (C) NE, not estimable

Best observed responses by RECIST 1.1 were 11 CR (19.3%), 23 PR (40.3%), 17 SD (29.8%), 3 PD (5.3%), and 3 non-evaluable cases (5.3%; table 3). Best percentage changes in target lesion size from baseline are displayed in figure 1. Median time to response was 5.7 months (95% CI 2.7 to 8.2). Figure 2 shows the duration of treatment and response of patients who experienced disease control. Median duration of response was not reached. Kaplan-Meier curves of PFS per RECIST 1.1 and OS are displayed in figure 3. Median survivals were not reached. The 12-month PFS and OS per RECIST 1.1 were 72.9% (95% CI 59.0% to 82.7%) and 84.0% (95% CI 71.4% to 91.3%), respectively.

### Parameters associated with efficacy

The MSI/dMMR status was centrally confirmed in 11 of 15 patients with a PFS event. Four tumor samples were not evaluable. Overall, eight patients had dMMR and MSI tumors and three pMMR and microsatellite stable (MSS) as assessed by central review. Of the latter three patients, one achieved PR of liver metastases, but had confirmed PD of the primary lesion 24 weeks after treatment initiation, and died due to disease-related event thereafter. The patients with MSS/pMMR mCRC were excluded from the Cox models analysis.

In univariate Cox model (table 4), a carcinoembryonic antigen (CEA) cutoff of 60 ng/mL (identified as the optimal cutoff by the restricted cubic splines method) was strongly associated with PFS (p=0.0025). The *KRAS*/*BRAF* mutational status tended to be associated with PFS (p=0.0973). Compared with patients who were wild type for *KRAS*/*BRAF*^V600E^, HR was 1.7 (95% CI 0.46 to 6.34) and 0.33 (95% CI 0.08 to 1.39) for those harboring *BRAF*^V600E^ and *RAS* mutations, respectively. In multivariate model, the CEA level of ≥60 ng/mL was the only baseline parameter associated with poorer PFS (HR=5.63, 95% CI 1.53 to 20.7, p=0.0094).

**Table 4:** Univariate and multivariate Cox regression analysis for progression-free survival

Factor	Univariate analysis					Multivariate analysis		
	Number	Number of events	HR	95% CI	P-value	HR	95% CI	P-value
Age (years)								
<65	43	8	1					
≥65	11	4	2.26	0.68 to 7.53	0.18			
Sex								
Male	29	5	1					
Female	25	7	1.82	0.58 to 5.74	0.31			
Body mass index								
≤20	14	4	1.58	0.44 to 5.59				
20–25	28	6	1		0.77			
25–30	8	1	0.527	0.06 to 4.38				
≥30	4	1	1.16	0.14 to 9.68				
Primary tumor location								
Right	29	8	1					
Left / rectum	24	4	0.556	0.17 to 1.85	0.34			
Grade								
Well differentiated	8	1	1					
Moderately differentiated	21	4	1.66	0.18 to 14.83				
Poorly differentiated	17	4	2.09	0.23 to 18.82	0.93			
Undifferentiated	1	0	–					
Stage								
II	4	1	1					
III	17	3	0.79	0.08 to 7.63				
IV	25	6	1.08	0.13 to 9.01	0.91			
Number of metastatic sites								
1–2	40	10	1					
>2	14	2	0.61	0.13 to 2.79	0.52			
Number of anterior therapeutic sequences								
1–2	28	5	1					
>2	25	6	1.54	0.47 to 5.04	0.48			
Antibiotics before inclusion								
No	45	9	1					
Yes	6	3	2.86	0.77 to 10.65	0.12			
Mutational status								
Wild type	18	5	1			1		
*BRAF*^V600E^-mutated	9	4	1.70	0.46 to 6.34		0.68	0.16 to 2.94	
*RAS*-mutated	27	3	0.33	0.08 to 1.39	**0.10**	0.32	0.07 to 1.34	0.30
Origin of MMR deficiency								
Sporadic	15	4	1					
Lynch-related	31	5	0.55	0.15 to 2.06	0.38			
Carcinoembryonic antigen (median)								
<12	28	4	1					
≥12	26	8	2.46	0.74 to 8.19	0.14			
Carcinoembryonic antigen*								
<60	41	5	1			1		
≥60	13	7	5.94	1.87 to 18.84	**0.002**	5.63	1.53 to 20.72	**0.009**
dNLR								
<3	37	7	1					
≥3	17	5	1.75	0.556 to 5.525	0.34			

N stands for number of (changed); P value stands for P-value (changed).

*Modeled with the restricted cubic splines method.

dNLR, a derived neutrophils/leukocytes minus neutrophils ratio; MMR, DNA mismatch repair.

## Discussion

In this open-label, multicenter, phase II study, the combination of nivolumab and ipilimumab was associated with a low frequency of pseudoprogression and high clinical activity in patients with MSI/dMMR mCRC.

This is the first report of the pseudoprogression incidence in a large group of patients with mCRC with MSI/dMMR tumors. Only two patients (3.5%) experienced pseudoprogression in our study, with the frequency lower than expected value. The incidence of pseudoprogression in previous studies of patients with melanoma, lung, or renal cancers did not exceed 10% with anti-PD1 monotherapy.^12 15 22 23^ Of note, this estimation is impaired by the fact that the definition of pseudoprogression varied across different studies.^24^ Here, we defined pseudoprogression an unconfirmed PD, per central review following the iRECIST guidelines, with CT scans performed every 6 weeks for 24 weeks.^14^ Even if one pseudoprogression was detected after 36 weeks of treatment in our study, it is thought to be an early phenomenon that might not be detected with a conventional interval of 8 weeks between tumor assessments.^25^ One might hypothesize that the frequency of pseudoprogression might vary depending on the type of ICI (anti-PD-L1 alone or in combination with anti-CTLA-4) used. However, data are lacking to make comparison of the pseudoprogression frequency in patients with MSI/dMMR mCRC treated with anti-PD-L1 alone, of which the associated pseudoprogression incidence is currently unknown, with those treated by anti-PD-L1 and anti-CTLA-4 agents. Still, our results suggest that RECIST 1.1 can be safely used as the primary criteria for response-based endpoints in randomized trials, especially those evaluating combinations of anti-PD1 and anti-CTLA-4 monoclonal antibodies versus conventional chemotherapy.

Of the 11 patients diagnosed with radiologic PD according to RECIST 1.1 criteria in our study, two had pseudoprogression (18.0%) and were still alive and free of progression at the last follow-up visit, whereas the remaining (82.0%) had confirmed PD of whom five died during follow-up. In other words, patients experiencing PD per RECIST 1.1 criteria while treated with nivolumab and ipilimumab are more likely to have confirmed PD and poor outcomes than pseudoprogression. Given the observed activity of ICI extended beyond toxicity-related treatment interruption, it might be more beneficial to promptly change antitumor treatment rather than maintain ICI until the next CT scan assessment. This should be discussed between the patient and their providers for a shared decision making when considering treatment continuation after PD.^17 26^

In this study with heavily pretreated patients, the combination of nivolumab and ipilimumab was associated with impressive efficacy and manageable toxicity. All 36 patients who completed the predefined 1 year of therapy were alive and free of progression at the last follow-up visit. The objective response rate was 59.6%, and the 12-month PFS and OS estimates were 72.9% and 84.0%, respectively. These results, with 1 year of immunotherapy, are consistent with previously published data on the combination cohort from the CheckMate-142 study, in which patients with controlled disease were mainly treated for longer than 2 years.^7^ Longer follow-up in this study is necessary to evaluate if the control of the disease continues after ICI treatment discontinuation (all patients received up to 1 year of therapy) and to determine median duration of response. Interestingly, the number of patients harboring disease resistance to immune checkpoint blockade with nivolumab and ipilimumab seems lower than with anti-PD-L1 monotherapy.^5 8–10^ In the KEYNOTE-177 phase III trial that demonstrated the superiority of pembrolizumab over first-line chemotherapy, 29.0% of patients in the experimental arm had PD by RECIST 1.1 as best observed response, while only 10.0% of patients in the NIPICOL study had PD or death as the best response.^10^ The CheckMate-8HW phase III study comparing nivolumab and nivolumab plus ipilimumab to chemotherapy should provide valuable data into this topic.

Given the durable activity of ICIs among responders, the main challenge from a clinical and scientific perspective will be to develop biomarkers that may predict the resistance of MSI/dMMR tumors to immunotherapy. We have reported, similarly to others, a significant amount of resistant cases related to a misdiagnosis of the MSI/dMMR status, with MSS/pMMR tumors incorrectly considered as MSI/dMMR.^27 28^ A review of pathological and molecular reports was therefore mandatory prior to the study enrollment. Despite that, a central reassessment detected three misdiagnosed patients (5.3%), who experienced PD during the study treatment. This observation highlights the importance of the accurate diagnosis of MSI/dMMR status in routine practice.

In addition to MSI/dMMR misdiagnosis, a CEA level of ≥60 ng/mL was the only factor significantly associated with decreased PFS, though this cutoff is more likely to be a negative prognostic factor than a predictor of resistance to immunotherapy. We acknowledge that, with only 15 PFS events, our analysis lacks of statistical power. Centrally assessed responses were observed irrespective of the mechanism underlying the MMR deficiency (sporadic vs Lynch-related), the *RAS*/*BRAF* status, and the primary tumor sidedness. Whereas results from the KEYNOTE-177 study suggested that the efficacy of pembrolizumab might vary depending on the *RAS* mutational status, our results are in line with previous reports demonstrating the clinical activity of ICIs whatever the disease subset.^5 7 10^ Other potential determinants of treatment efficacy including the use of antibiotics at baseline,^29 30^ the derived neutrophils/(leukocytes minus neutrophils) ratio,^31^ or the body mass index^32^ did not predict patients’ outcomes in our study. Other small cohort studies have shown that tumor mutational load and immune infiltrate are interesting parameters deserving further research.^27 33 34^ Translational studies with tumor samples from this trial are currently evaluating potential biological predictive factors.

In summary, this phase II study confirms high activity of nivolumab plus ipilimumab for four cycles followed by nivolumab alone for maximum 1 year of therapy in patients with MSI/dMMR mCRC. The ongoing CheckMate-8HW phase III study should provide useful information on the clinical benefit of nivolumab plus ipilimumab versus nivolumab alone in this setting. In patients with MSI/dMMR who exhibit PD per RECIST 1.1 criteria, the likelihood of pseudoprogression is low compared with the risk of real progression. Close clinical monitoring and shared information with patients are required if treatment continues beyond progression.
