# Supplementary material for: RECIST and iRECIST criteria for the evaluation of nivolumab plus ipilimumab in patients with microsatellite instability-high/mismatch repair-deficient metastatic colorectal cancer: the GERCOR NIPICOL phase II study
Source: J Immunother Cancer. 2020 Nov 3;8(2):e001499. doi: 10.1136/jitc-2020-001499 (PMC7640587; doi:10.1136/jitc-2020-001499)
Supplement: Supplementary data [file jitc-2020-001499supp001.pdf]

Supplementary Table 1. Tumor response at 12 weeks and overall best observed response per iRECIST criteria

	**At 12 weeks** **N = 57**	**Overall best response** **N = 57**
Complete response, *n* (%)	2 (3.5)	11 (19.3)
Partial response, *n* (%)	18 (31.6)	23 (40.4)
Objective response rate, %	35.1	59.6
Stable disease, *n* (%)	30 (52.6)	18 (31.6)
Progressive disease, *n* (%)	4 (7.0)	2 (3.5)
Non evaluable, *n* (%)	3 (5.3)	3 (5.3)

Cohen R, *et al. J Immunother Cancer* 2020; 8:e001499. doi: 10.1136/jitc-2020-001499
